# From SilverStar to shining stars: Physiology's next generation

**DOI:** 10.1113/EP093871

**Published:** 2026-04-21

**Authors:** Damian M Bailey

**Affiliations:** ^1^ Neurovascular Research Laboratory, Faculty of Life Sciences and Education University of South Wales Glamorgan UK

Early career researchers (ECRs) are not simply the future of physiology; they are its living, working present. They bring intellectual energy, technical innovation and the courage to ask new questions at a time when our discipline is expanding across scales, systems and settings. Yet to celebrate ECRs without acknowledging the headwinds they face would be disingenuous. This editorial reflects on both sides of the equation. It considers the mounting challenges confronting ECRs in physiology but also reflects on a recent experience that left me genuinely inspired: attendance at the seventh biennial Okanagan Cardiovascular & Respiratory Symposium, held at SilverStar Mountain Resort, British Columbia, Canada, from 12 to 14 March 2026 (Figure 1). This meeting, run by the Centre for Heart, Lung and Vascular Health at the University of British Columbia Okanagan, provided a striking example of what can happen when ECRs are not treated as an afterthought, but instead placed deliberately and unapologetically at the very core of a scientific meeting. Although the majority of ECRs came from Canada and the USA, the growing presence of delegates from Europe and Australasia highlighted the expanding international profile of the symposium. Importantly, financial support from *Experimental Physiology* and *The Journal of Physiology*, together with faculty and industry support, kept ECR attendance fees manageable (<$100, including food), improving accessibility for those who might otherwise have been priced out, while also supporting the Dr Chris Willie Graduate Prize for Excellence in Research.

**FIGURE 1:**
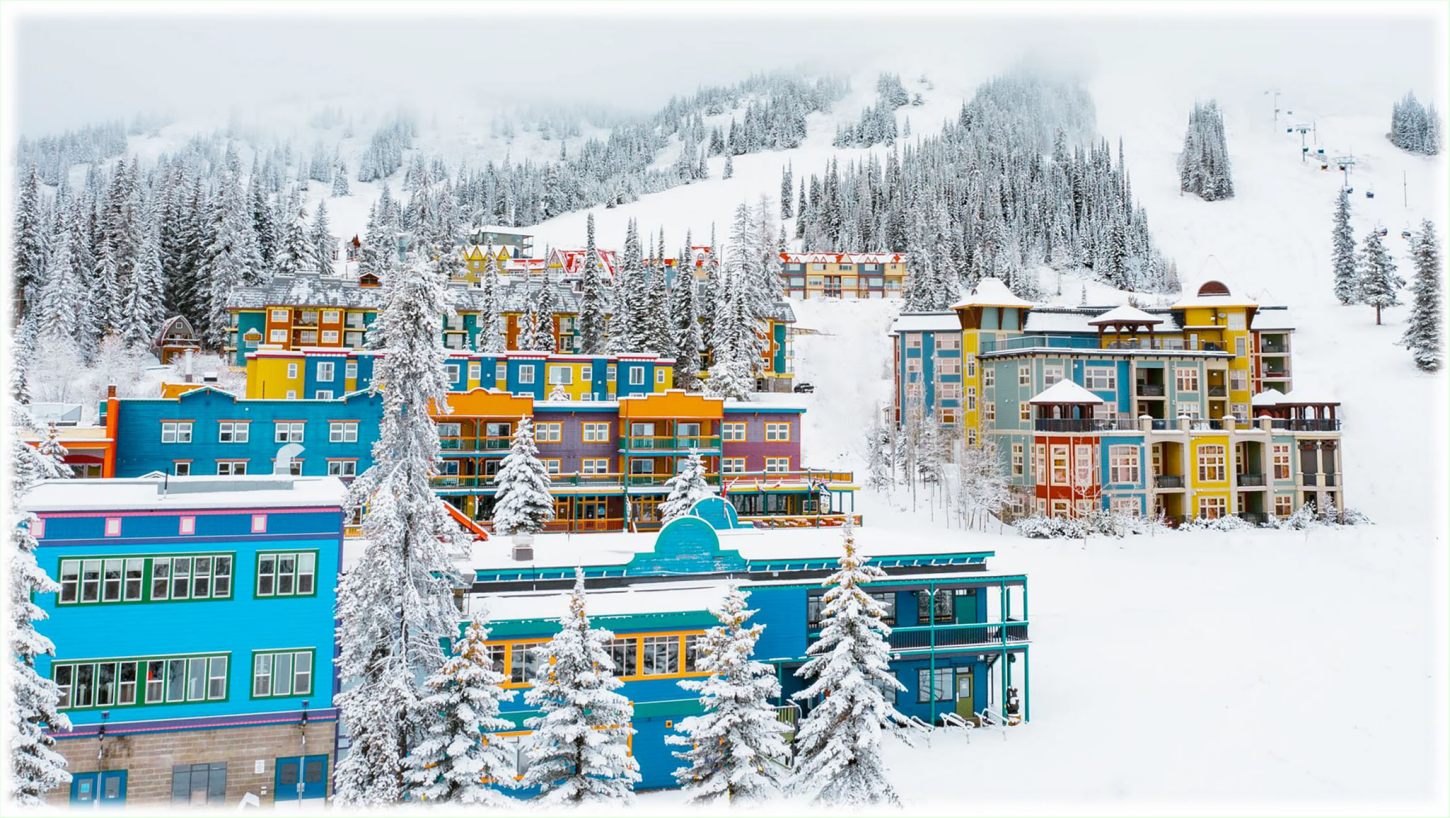
SilverStar ski resort in British Columbia where the biennial Okanagan Cardiovascular & Respiratory Symposium is held. Photograph credit: Philippa Coates.

Life as an ECR in physiology is far from easy. From doctoral candidates and postdoctoral fellows to newly independent principal investigators, this group constitutes one of the largest and most diverse segments of the research workforce, yet remains among the most professionally vulnerable (Nature, 2020; Nicholls et al., 2022). These longstanding academic pressures have been amplified in the post‐pandemic era by short‐term contracts, intense competition for funding, escalating expectations for publication and impact, and persistent uncertainty over whether present sacrifice will yield future stability. Job insecurity, coupled with the demands of academic life, has consequently emerged as a major threat to mental health and wellbeing (Nicholls et al., 2022). *Nature*’s global survey of postdoctoral researchers captured this unease starkly, revealing widespread anxiety over career prospects, long working hours and poor mental health, with many questioning whether they would choose the same path again (Woolston, 2020).

More recent analysis of these survey data sharpens the picture by showing that the balance between job demands and job resources is critical: longer working hours, frequent overtime and job insecurity were associated with poorer work–life balance and a greater likelihood of mental health difficulties, whereas mentor support, job autonomy and institutional reward structures appeared to buffer these risks (Yang et al., 2025). Notably, these effects were mediated through work–life balance satisfaction, reinforcing the argument that healthier academic environments are not a luxury for ECRs, but a prerequisite for sustaining both wellbeing and scientific productivity. Together, these findings underscore broader warnings that academia risks losing talented ECRs unless more sustainable and supportive career structures are put in place (Langin, 2022; Nature, 2020).

For too many, the problem is not ability but architecture. ECRs spend years suspended between promise and permanence, moving from one temporary contract to the next, often relocating countries, rebuilding collaborations and delaying financial and personal stability in the process. They are expected to teach, publish, review, supervise, network and secure funding, all while constructing their own independent scientific identity. It is an exhausting paradox: they are asked to think boldly but often employed precariously. The result is that a career stage that ought to be defined by curiosity, creativity and development can instead become dominated by fragility and a fight for survival. In the UK, only a small minority of doctoral researchers will ultimately progress into permanent academic posts, a reality that should give pause to everyone who depends upon their talent, labour and imagination.

Physiology, perhaps more than most disciplines, should be especially alert to this problem. Ours is an integrative science that thrives on apprenticeship, discussion, experimentation and conceptual cross‐fertilization. ECRs do not merely populate the pipeline; they are the workhorses of research‐intensive institutions, driving the productivity and innovation needed to sustain future scientific leadership, while actively shaping the questions we ask, the methods we deploy, and the translational reach of the field. If they are lost to disillusionment, then physiology loses more than personnel; it loses momentum, diversity of thought, and scientific possibility.

Against that backdrop, my recent experience at the Okanagan Cardiovascular & Respiratory Symposium was especially refreshing. This was not a meeting where ECR talks were squeezed into the least desirable slots or treated as a prelude to the ‘main event’. Far from it, they were the main event! The symposium, held over 3 days in a truly memorable mountain setting in SilverStar ski resort located just outside of the town of Vernon in British Columbia between 1155 and 1915 m a.s.l., revolved around giving ECRs the spotlight in an informal, constructive and intellectually stimulating environment. Morning and evening sessions featured world‐renowned principal investigators covering diverse aspects of integrative physiology, including thermal physiology, sex differences in cerebrovascular control, new insights into the oxygen cascade, comparative physiology and native high‐altitude adaptation. But the dominant rhythm of the meeting belonged to the ECRs, whose talks, ranging from concise 6 min snapshots to more developed 12 min, drove the science and the discussion. The quality of this work was further reflected in the student prize winners (Figure 2), with the MSc Award presented to Nicole Bushfield for ‘Integrated physiologic and proteomic signatures of post‐cardiac arrest brain injury’ (supervised by Dr Ryan Hoiland) and the PhD Award to Bryony Curry for ‘Enhanced stroke volume and divergence of the human left ventricle’ (supervised by Professor Rob Shave).

**FIGURE 2:**
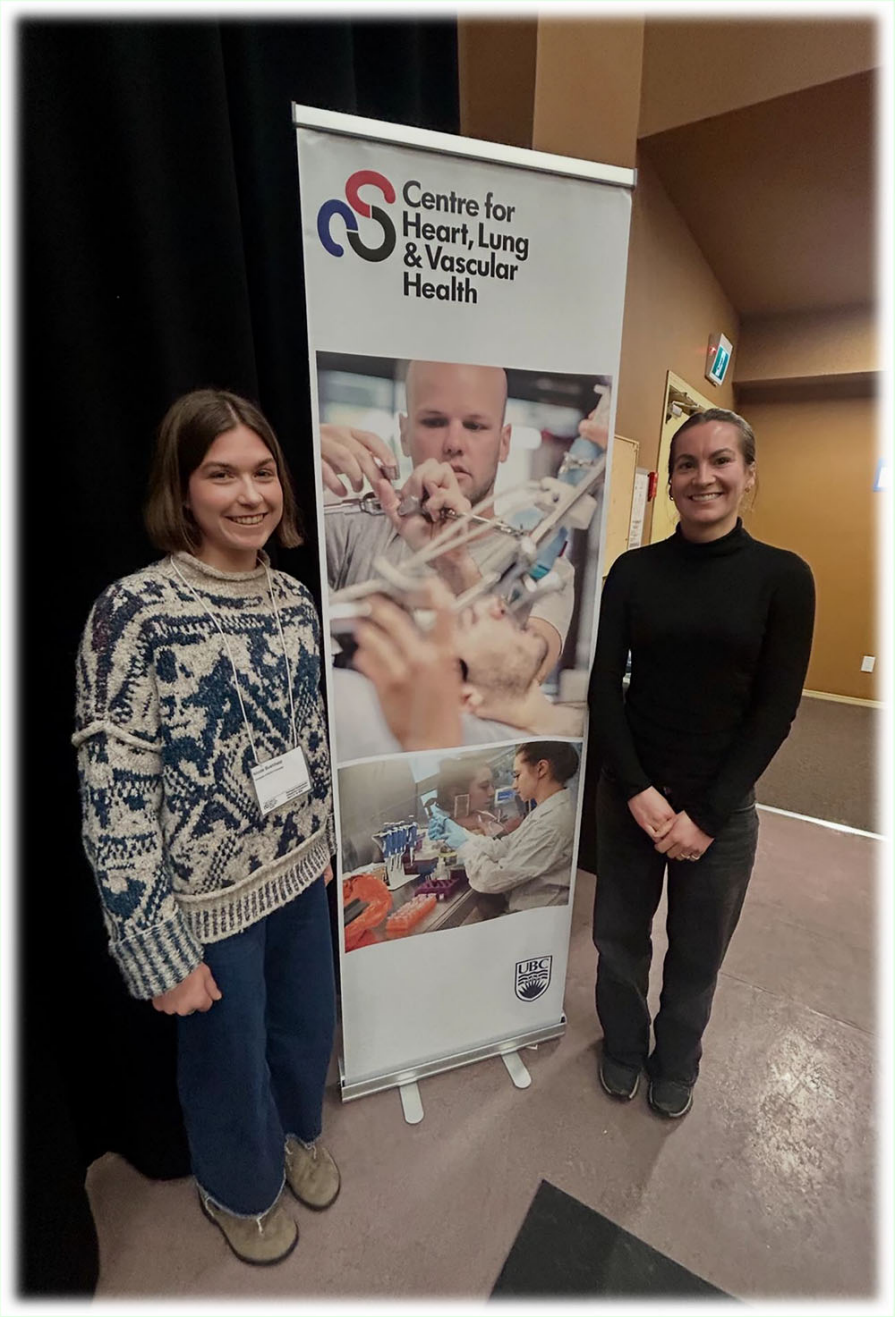
Student prize winners. Left, Nicole Bushfield MSc Award; right, Bryony Curry PhD Award. Photograph credit: Professor Neil D. Eves.

What struck me most was not simply the quality of the research, although that was uniformly impressive. It was the atmosphere in which that research was allowed to ‘breathe’. The meeting created a space in which ECRs could showcase their data with supervisors and principal investigators in the room, yet without the oppressive sense that they were being judged from above. Questions were probing yet constructive; discussion was rigorous yet supportive. There was an unmistakable sense that the purpose of the meeting was not to expose weakness, but to refine ideas, build confidence and accelerate development. This matters, because effective mentorship is not a vague academic virtue, but a practical framework that helps ECRs to align expectations, strengthen communication, navigate equity and inclusion, and develop a clearer professional identity and direction (Diggs‐Andrews et al., 2021). Just as importantly, no single mentor can provide all these functions; ECRs benefit most from mentoring networks that combine established mentors with peer support, offering complementary scientific, professional and psychosocial guidance (Diggs‐Andrews et al., 2021). In this respect, SilverStar did more than showcase excellent ECR science: it fostered exactly the kind of layered mentoring environment that current best practice would advocate.

This collegial spirit extended beyond the formal sessions into the shared meals, where all delegates ate together and where established principal investigators and keynote speakers would actively seek out ECRs to continue scientific discussions. For many, this provided a particularly valuable opportunity to network, build confidence and seed future collaborations. The science itself reflected the breadth and richness of integrative physiology, spanning thermal, high‐altitude, exercise and comparative physiology, often with a translational twist. Just as importantly, several presenters explicitly identified opportunities for collaboration, recognizing that the next leap in their science might depend on combining conceptual approaches and measurement capabilities across groups and disciplines. A friendly and inclusive call for collaboration!

That is precisely why meetings like this matter. Nurturing ECRs is not a soft exercise in encouragement or line item on a curriculum vitae; it is a hard‐headed investment in better science. We should want our ECRs to encounter challenge, but in environments where challenge is coupled to mentorship, visibility and opportunity. We should want them to learn that collaboration is not a sign of weakness or incompleteness, but one of the hallmarks of integrative physiology. No laboratory, however strong, owns all the tools or all the answers. When ECRs are exposed early to different scientific cultures, different measurement capabilities and different ways of thinking, their physiology becomes more integrative, more adventurous and, ultimately, more impactful.

The SilverStar format also underscored something else that academia sometimes neglects: relationships matter. Parts of the day were deliberately set aside for skiing (!), networking and social events, and these were not peripheral indulgences. They were integral to the pedagogy. Science advances through trust, conversation, generosity, and the confidence to float an unpolished, often embryonic idea before it is publication ready. A memorable setting does not replace intellectual substance, but it can create the conditions in which intellectual substance is more freely exchanged. In that sense, the symposium offered a powerful reminder that the best meetings are not simply platforms for data exchange; they are ‘ecosystems’ for confidence‐building, community formation and scientific imagination underpinned by exploration and discovery. This sense of shared purpose and community is captured nicely in the group photograph taken at the National Altitude Training Centre where the meeting was held (Figure 3).

**FIGURE 3:**
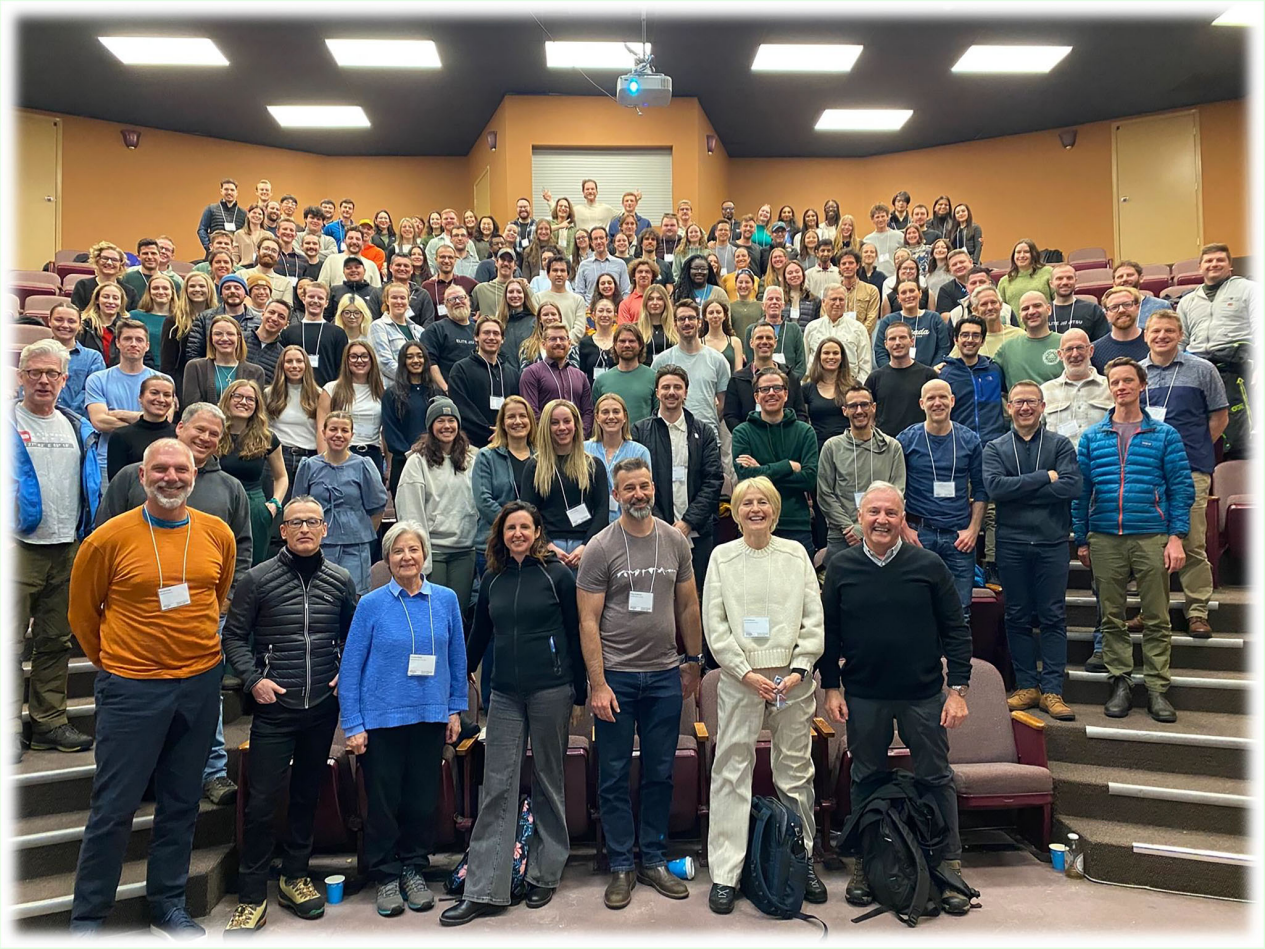
Group photograph. Photograph credit: Professor Neil D. Eves.

Drs Neil Eves and Philip Ainslie, together with Mrs Audrey Kirby, deserve warm congratulations for creating and sustaining such a meeting. The time, effort and careful organization required to deliver an event of this quality are far from insignificant, which is precisely why it is held only biennially. The result, however, is one of the true highlights of the international physiology calendar, and the team warrants sincere recognition for maintaining a forum that continues to inspire both established investigators and our next‐generation ECRs. They have delivered a pedagogic, equitable and inclusive model that conference organizers, learned societies and university departments alike should study carefully. Its impact was plain to see. I will be fascinated to observe how this meeting shapes the trajectories of the ECRs who attended, many of whom will surely emerge as future leaders in physiology. For my own part, it renewed a conviction that we would do well to remember more often: research training should be shaped less by managerial process and more by active researchers who understand that science begins, and continues, with ideas. At SilverStar, I was privileged to witness a remarkable concentration of exactly that: bold, creative, cutting‐edge ideas, carried by the very people in whom the future of physiology now rests. I, for one, am already looking forward to the eighth meeting in 2028.

## AUTHOR CONTRIBUTIONS

Damian M. Bailey conceived the idea and wrote the first draft of the manuscript. Damian M. Bailey approved the final version submitted for publication and agrees to be accountable for all aspects of the work in ensuring that questions related to the accuracy or integrity of any part of the work are appropriately investigated and resolved. All persons designated as authors qualify for authorship, and all those who qualify for authorship are listed.

## CONFLICT OF INTEREST

D.M.B. is Editor‐in‐Chief of *Experimental Physiology* and outgoing Chair of the Life Sciences Working Group and outgoing member of the Human Spaceflight and Exploration Science Advisory Committee to ESA. D.M.B. is a current member of the ESA‐HRE‐Biology Panel and Space Exploration Advisory Committees to the UK and Swedish National Space Agencies.
